# Case Report of a Prostatic Abscess with a Review of the Literature

**DOI:** 10.1155/2012/430657

**Published:** 2012-08-30

**Authors:** Michael T. Flannery, Deborah Humphrey

**Affiliations:** Morsani College of Medicine, University of South Florida, Tampa, FL 33612, USA

## Abstract

A middle age male with type 2 diabetes mellitus presented with urinary symptoms. Blood and urine cultures were consistent with Methicillin Resistant *Staphylcoccal Aureus* (MRSA). A computed tomography demonstrated mutiple prostatic microabscessess. No other hematogenous source was identified. Transurethral prostatic drainage and intravenous Vancomycin followed by oral doxcycline led to clinical success for this likely Community acquired case of MRSA (CA-MRSA). We discuss our case report and discuss the current literature on the trends, causation, diagnosis and treatment of MRSA induced prostatic abscess.

## 1. Introduction 

While MRSA infections are increasing in our communities, unusual sites of involvement continue to be reported especially in those patients with underlying risk for immunosuppression. Our case involves a patient with Type 2 diabetes mellitus who presented with nonspecific abdominal symptoms with blood cultures positive for MRSA. Computed tomography confirmed multiple prostatic abscesses which required appropriate drainage and antibiotic therapy to achieve clinical success.

## 2. Case Presentation

**Figure 1:**
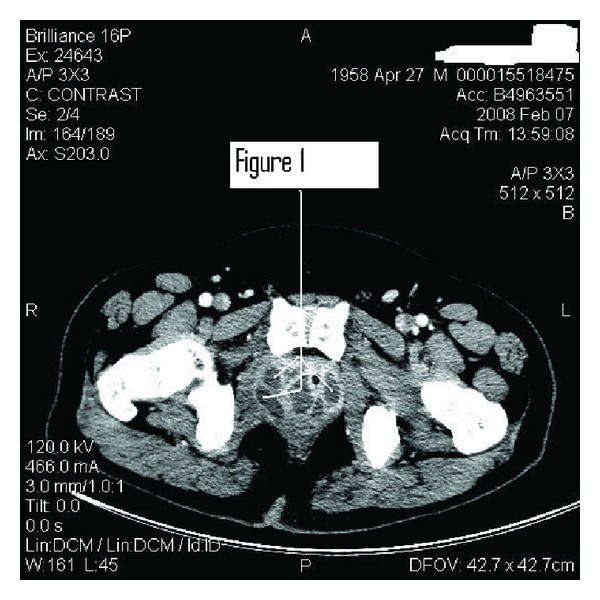
Computed tomography of the pelvis demonstrating multiple prostatic abscesses of varying size.

A 49-year-old Hispanic male with fairly well-controlled type two diabetes mellitus presented with several days of subjective fever with difficult urination including micturition of small amounts and dribbling associated with low back pain. His exam demonstated mild tachycardia with no fever and normal blood-pressure measurements. His skin was clammy with suprapubic tenderness and bilateral mild low back pain with no vertebral tenderness. The prostate was 3+ and boggy with significant tenderness, The laboratory studies were only significant for leukocytosis of 16.2 × 10^9^ L with a bandemia of 10%. His labs were otherwise normal except for a creatinine of 114.9 *μ*mol/L and a glucose of 14.5 mmol/L. The prostatic-specific antigen (PSA) was 3.1 ug/L prior to therapy. Testing for the human immunodeficiency virus and chlamydial/gonorrheal urinary antigens were negative. A Foley was placed, and the urinary sediment showed significant leucocytes with moderate protein and a small amount of blood. His chest X-ray was normal. A computed tomography of the abdomen and pelvis demonstrated a large prostate with hypodensities consistent with prostate abscesses (Figure 1). The patient was started on intravenous fluids and antibiotics with glucose control via a sliding scale of regular insulin. The blood cultures and urine culture all grew Methicillin Resistant *Staphylcoccus Aureus* (MRSA). Vancomycin was added to his regimen but clinically and radiographically and, not unexpectedly, he did not improve so he underwent a transurethral resection of the prostate (TURP) with abscess drainage. The pathologic specimen demonstrated benign prostatic tissue with severe acute, necrotizing prostatitis. With intravenous Vancomycin and drainage the patient markedly improved clinically with negative cultures of the urine and blood. The Foley remained in place for two weeks and then was pulled without difficulty. He was subsequently treated with another month of oral doxycycline with clinical success. A six- week followup PSA level was 1.2 ug/L.

## 3. Discussion

There is an increasing number of reports of MRSA associated with prostatic abscess [1–4]. A number of different theories exists regarding causation which includes the following.

1. Retrograde flow of contaminated urine during micturation.
2. Prostatitis, acute or chronic.
3. Hematogenous spread from distant foci including skin, respiratory, and gastrointestinal sites.

Currently, *Escherichia coli* are the most common cause of prostatic abscess with a history of *Neisseria gonorrhea* and *Enterobacter* dominance in prior decades. Predisposing factors for prostatic abscess include diabetes mellitus, chronic bacterial prostatitis, instrumentation of the lower urinary tract, indwelling catheters, intravenous drug use, human immunodeficieny virus, and bladder outlet obstruction [2]. A number of factors have contributed to an etiologic shift such as improved patient care which has increased longevity, and therapeutic advances in transplantation, hemodialysis and chemotherapy. 

Our patient had diabetes as a risk factor but no other sources identified by testing for hematogenous spread. Once obtained by culture, our patient was treated by intravenous Vancomycin, and, given its hematogenous spread, treatment was continued intravenously for two weeks. If patients are intolerant or allergic to Vancomycin there has been a reported case of a prostatic abscess in a diabetic successfully treated with Daptomycin [4]. Subsequent to intravenous therapy, most authors subsequently recommend treatment with drugs that have good prostate penetration for another month orally. These include sulfa, fluoroquinolones and tetra or doxycycline given their good penetration and activity of against MRSA. Of course drainage is recommended for the majority of prostatic abscesses, however, TURP or transrectal or perineal drainage may be associated with transient further hematogenous spread.

Obviously, cases may involve both hospital and community acquired MRSA. Community acquired MRSA (CA-MRSA) infection is defined as MRSA infection occurring in the community or <48 h after hospital admission in patients with no history of admission to a hospital or a nursing home in the past year. Community-acquired MRSA is not associated with a history of dialysis, surgery, permanent indwelling catheters, or medical devices that pass through the skin into the body [2]. Community-acquired versus hospital acquired species of MRSA is genotypically separate with the majority of the community-resistant strains identified as USA 300 [4].

In our case, it would have been assumed that the infection was gram negative in origin, and the patient would have failed therapy if the blood and urine cultures had not come back with consistent evidence of MRSA. Our patient's symptoms, urinary sediment and cultures all improved with Vancomycin. After subsequent additional therapy for one month with doxycycline, the patient was doing well. Our patient also met the criteria for CA-MSA. It has been demonstrated that CA-MRSA presents 8–20% of all MRSA isolates in the United States of America [5]. It is certainly important to consider MRSA in patients with predisposing conditions, and to consider the source of the infection. 

In summary, we present a type 2 diabetic who likely developed CA-MRSA of the prostate which required appropriate antibiotic therapy without clinical improvement or clearance of his blood cultures. Given his multiple abscesses, drainage with a TURP was important in achieving clinical success. In patients with risk factors for MRSA, gram staining of the urinary sediment while awaiting culture results is vital in determining appropriate initial antibiotic therapy.
